# The coordination of ploidy and cell size differs between cell layers in leaves

**DOI:** 10.1242/dev.130021

**Published:** 2016-04-01

**Authors:** Yohei Katagiri, Junko Hasegawa, Ushio Fujikura, Rina Hoshino, Sachihiro Matsunaga, Hirokazu Tsukaya

**Affiliations:** 1 Department of Applied Biological Science, Faculty of Science and Technology, Tokyo University of Science, 2641 Yamazaki, Noda, Chiba 278-8510, Japan; 2 Department of Biological Sciences, Graduate School of Science, The University of Tokyo, Bunkyo-ku, Tokyo 113-0033, Japan; 3 Bio-Next Project, Okazaki Institute for Integrative Bioscience, National Institutes of Natural Sciences, Yamate Build. #3, 5-1, Higashiyama, Myodaiji, Okazaki, Aichi 444-8787, Japan

**Keywords:** Endoreduplication, Ploidy, Cell volume, Mesophyll tissue, Epidermis, *ATML1*

## Abstract

Growth and developmental processes are occasionally accompanied by multiple rounds of DNA replication, known as endoreduplication. Coordination between endoreduplication and cell size regulation often plays a crucial role in proper organogenesis and cell differentiation. Here, we report that the level of correlation between ploidy and cell volume is different in the outer and inner cell layers of leaves of *Arabidopsis thaliana* using a novel imaging technique. Although there is a well-known, strong correlation between ploidy and cell volume in pavement cells of the epidermis, this correlation was extremely weak in palisade mesophyll cells. Induction of epidermis cell identity based on the expression of the homeobox gene *ATML1* in mesophyll cells enhanced the level of correlation between ploidy and cell volume to near that of wild-type epidermal cells. We therefore propose that the correlation between ploidy and cell volume is regulated by cell identity.

## Abstract (precis)

**Summary:** The well-known correlation between ploidy and cell volume in the leaf epidermis is not seen in leaf palisade mesophyll cells, indicating that cell identity influences this correlation.

## INTRODUCTION

Ploidy levels are closely associated with cell size in many organisms (Mendell et al., 2008; Fox and Duronio, 2013; Matsunaga et al., 2013). Increases in the ploidy level in somatic cells are accomplished through endoreduplication, which is also known as endoreplication or the endocycle. Specifically, the cell cycle skips the mitotic phase, including the segregation of sister chromatids or chromosomes after DNA replication, resulting in polyploid cells. Rapid DNA replication is believed to contribute to the activation of metabolism for cell growth and differentiation, as well as resistance to DNA damage (Adachi et al., 2011) and parasitic infections (Vieira et al., 2013).

Studies using the model plant *Arabidopsis thaliana* have shown that a high ploidy level caused by endoreduplication is reflected by enhanced cell expansion (Breuer et al., 2010). For example, differentiation and morphogenesis of large, single-celled trichomes (Hülskamp et al., 1999), extensive elongation of hypocotyls under dark conditions (Jakoby and Schnittger, 2004) and the differentiation of giant cells in the sepal epidermis (Roeder et al., 2010) depend on enhanced cell expansion by endoreduplication. Furthermore, in pavement cells of the leaf epidermis, the distribution of cell size correlates directly with ploidy level (Melaragno et al., 1993), indicating that cell size is under the control of endoreduplication. However, this universal correlation between ploidy and cell size seems to have been overestimated in *A. thaliana*. First, because of the difficulty in making observations at the single cell level in the inner cell layers, measurements of cell size and ploidy levels by microscopy have only examined the epidermal cells in the outermost L1 layer. All previous measurements of the size of palisade mesophyll cells in *A. thaliana* mutants and transgenic plants indicate that the palisade mesophyll cells did not show a ploidy correlated, multi-peak distribution pattern for size, as observed in epidermal cells (Tsuge et al., 1996; Kim et al., 1998; Horiguchi et al., 2006; Ferjani et al., 2007). However, our previous measurements provided a mean cell size with a small standard deviation of the palisade mesophyll cells (Kim et al., 1998; Horiguchi et al., 2006; Ferjani et al., 2007; Fujikura et al., 2007).

Conventional flow cytometry to detect endoreduplication is typically performed on leaf segments and data on the level of endoreduplication are thought to be predominantly for the inner tissues (because the proportion of epidermis is low compared with inner tissue), strongly suggesting that the inner tissues also exhibit extensive endoreduplication. However, many previous studies suggest that the relationship between the ploidy level and cell size is not always as simple as in *A. thaliana* epidermis. For example, the relationship between the ploidy level and cell size in sepals is not necessarily linear (Roeder et al., 2012). Bourdon et al. (2011) also suggest that cell size is not only dependent on ploidy levels but also upon the position of the cell within the tissue according to an analysis of tomato pericarp. Moreover, whole-genome tetraploidization experiments showed that the size of tetraploid cells is not always twice the volume of diploid cells in palisade tissues and pollen grains (Tsukaya, 2013). Instead, some genetic regulatory systems are believed to control ploidy-dependent cell enlargement.

In this study, we measured the ploidy levels and size of leaf palisade mesophyll cells of *A. thaliana*. A combination of a new *in situ* imaging technique and genetic analysis revealed that cell identity regulates the relationship between ploidy level and cell size.

## RESULTS AND DISCUSSION

### A new technique enables optical measurement of the ploidy level in inner leaves

**Fig. 1.**
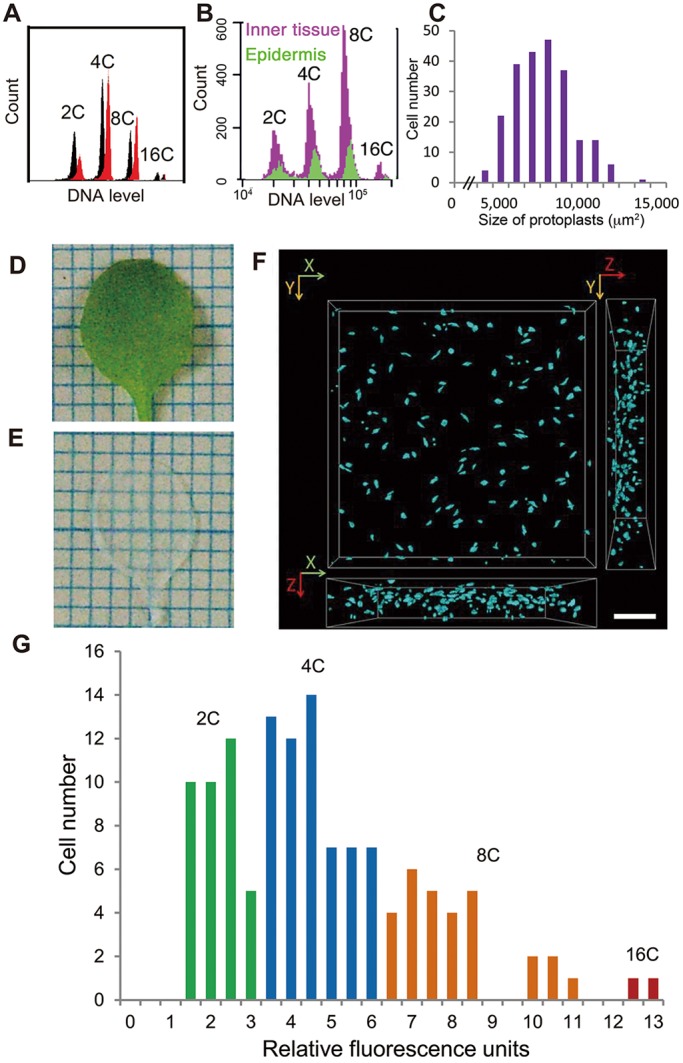
**A novel tissue-clearing technique enables analysis of ploidy levels in mesophyll cells.** (A) Ploidy levels obtained from whole-leaf samples of wild-type (WT) leaves (shown in black) and from mesophyll protoplasts of the same WT leaves from which the epidermis was peeled off (in red), respectively. Flow cytometry analysis was performed using 3-week-old first leaves. Typical ploidy distribution patterns from three independent trials are presented (*n*>102 cells). (B) Flow cytometry data on the first set of leaves collected from 3-week-old pATML1::H2B-mGFP plants. In this line, as reported previously (Roeder et al., 2010), nuclei from epidermis (shown in green) could be distinguished using GFP markers from those from the inner tissues (shown in purple), and allowed us to compare the endoreduplication level between epidermis and inner tissues. (C) Size distribution of protoplasts from the first set of leaves of 3-week-old plantlets. Values represent the projected area of protoplasts under the microscope. (D,E) The first foliage leaf of 30-day-old WT before (D) and after (E) transparency treatment. Grid squares equal 1 mm^2^. (F) 3D view of nuclei stained with DAPI in 30-day-old WT leaves. Scale bar: 50 μm. (G) Histogram of relative fluorescence units and cell numbers in WT palisade mesophyll cells from 10 first foliage leaves of 30-day-old WT plants. Each bar indicates 2C (green), 4C (blue), 8C (orange) and 16C (red).

First, the ploidy levels of inner mesophyll protoplasts were compared with conventional data obtained from whole leaf tissues without removing the epidermis for the first set of foliage leaves of Columbia wild-type (WT) *A. thaliana*. On the basis of flow cytometry data, leaves (Fig. 1A, black) and inner mesophyll protoplasts (Fig. 1A, red) showed a similar ploidy distribution pattern. Flow cytometry data on leaves of an epidermis-specific nuclear-tagged line, pATML1::H2B-mGFP (Roeder et al., 2010), also clearly indicated that both epidermis (Fig. 1B, green) and inner tissue (Fig. 1B, purple) exhibit endoreduplication at a similar level. By contrast, a single-peak distribution was observed for the size of mesophyll protoplasts (Fig. 1C). This was suggestive of a different relationship between ploidy level and cell size in mesophyll compared with epidermal cells. To analyse this relationship in mesophyll cells, a novel technique that renders tissues transparent was developed to avoid scattering light and autofluorescence from cell walls and chloroplasts by treating the samples with 2,2′-thiodiethanol after fixation (Fig. 1D,E). This new optical technique enabled deep imaging using normal confocal microscopy into the inner multicellular layers in clarified plant structures. Our method (termed TOMEI, for transparent plant organ method for imaging) is unique in that transparent leaves are prepared rapidly in only 3-4 h, compared with previous techniques, which may take several days or weeks (Warner et al., 2014; Kurihara et al., 2015). This method is also applicable to other species and organs (Hasegawa et al., 2016). By staining DNA with DAPI in combination with 3D analysis software, DNA content was quantitatively measured as an integrated intensity based on DAPI fluorescence units per cell nucleus. We used the 30-day-old first set of foliage leaves of WT plants, where growth stopped at the mature stage (Fig. 1F). The relative fluorescence unit is a doubling value obtained by dividing the integrated intensity of the nucleus by the average value of the integrated intensity from the nucleus of five guard cells. We counted the cell number per relative fluorescence unit and classified cells into four ploidy levels (2C to 16C; Fig. 1G). This histogram again indicated that palisade mesophyll cells in a subepidermal layer show extensive endoreduplication.

### Different correlation levels between ploidy and cell volume in the leaf layers

**Fig. 2.**
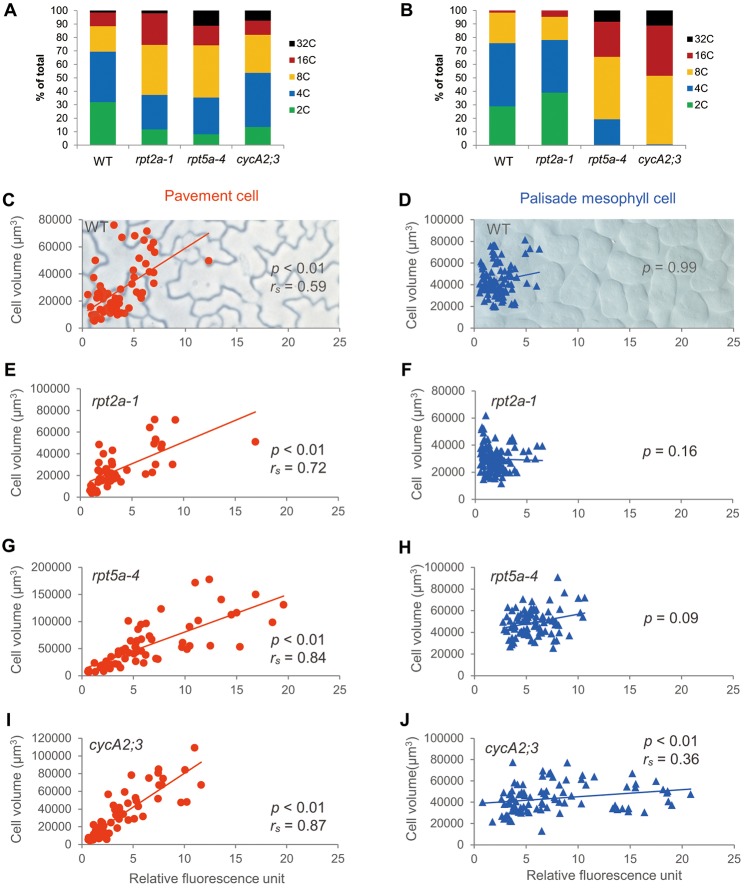
**The progression of endoreduplication varied between tissues.** (A,B) Ratio of ploidy levels in pavement cells (A) and palisade mesophyll cells (B) in WT and mutants examined here. (C-J) Relative fluorescence units and cell volume for individual epidermal cells (C,E,G,I) and palisade mesophyll cells (D,F,H J) in 30-day-old first foliage leaves of WT (C,D), *rpt2a-1* (E,F), *rpt5a-4* (G,H) and *cycA2;3* (I,J) plants. *r_s_* indicates the Spearman rank correlation coefficient. Data were collected from at least three different samples, and at least 50 pavement cells and 84 palisade mesophyll cells were analysed. The statistical results are summarized in Table 1.

Next, the relationship between ploidy level and cell volume in the pavement cells of the epidermis and palisade mesophyll cells was analysed based on optical measurements of the transparent leaves. Here, we calculated the Spearman rank correlation coefficient as a non-parametric measure of correlation (Fig. 2C,D, Table 1). In pavement cells, the correlation between ploidy and cell volume was strong (*P*<0.01, *r_s_*=0.59), but not significant in the palisade mesophyll cells (*P*=0.99). Moreover, the dispersion of cell volume at a given ploidy level was larger in palisade mesophyll cells than in the epidermis. Each leaf was analysed separately to demonstrate the reproducibility of the correlation in independent samples (Fig. S1, Table S1). This indicates that the relationship between cell volume and ploidy could be dependent on cell identity.

**Table 1.**
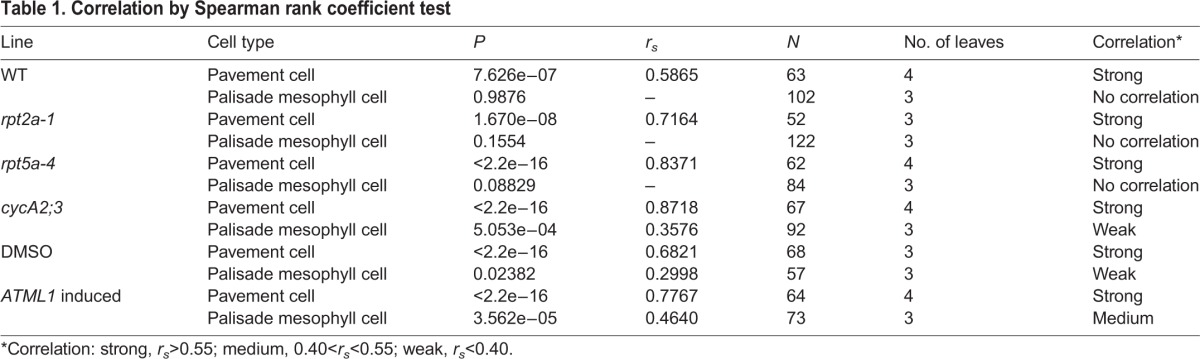
**Correlation by Spearman rank coefficient test**

The degree of ploidy dependency on cell size is known to be affected by genetic mutations during whole-genome tetraploidization (Breuer et al., 2007; Tsukaya, 2008, 2013). To explore whether this is also the case for endoreduplication-dependent cell volume control, some mutants with enhanced endoredupliation were measured using the tissue-clearing technique (Fig. 2A,B). RPT2a and RPT5a belong to the AAA ATPase family of the 26S proteasome regulatory particle (Sonoda et al., 2009; Sako and Yamaguchi, 2010), and CYCA2;3 is a key regulator of the endocycle (Imai et al., 2006). To compare ploidy dependency in the control of cell size, the correlation was calculated based on the *C*-value and the cell volume in each cell (Fig. 2C-J, Fig. S1, Table 1, Table S1). The correlation between ploidy and cell volume in pavement cells differed between WT and mutants, and also between palisade mesophyll and pavement cells. At higher ploidy levels, the correlation seemed to be somewhat clear, even in palisade mesophyll cells. An important point is that palisade mesophyll cells had a larger basal cell volume than that of pavement cells (compare Fig. 2D,F,H,J with C,E,G,I). In other words, the correlation between ploidy and cell volume is weaker at higher ploidy levels in palisade mesophyll cells because of the presence of a larger ‘basal’ cell volume that is independent of the ploidy level. If so, cell volume could be divided into ‘basal’ and ploidy-dependent parts; the former is smaller in the epidermis and larger in mesophyll cells.

### Cell identity of leaves determines the volume of each cell

**Fig. 3.**
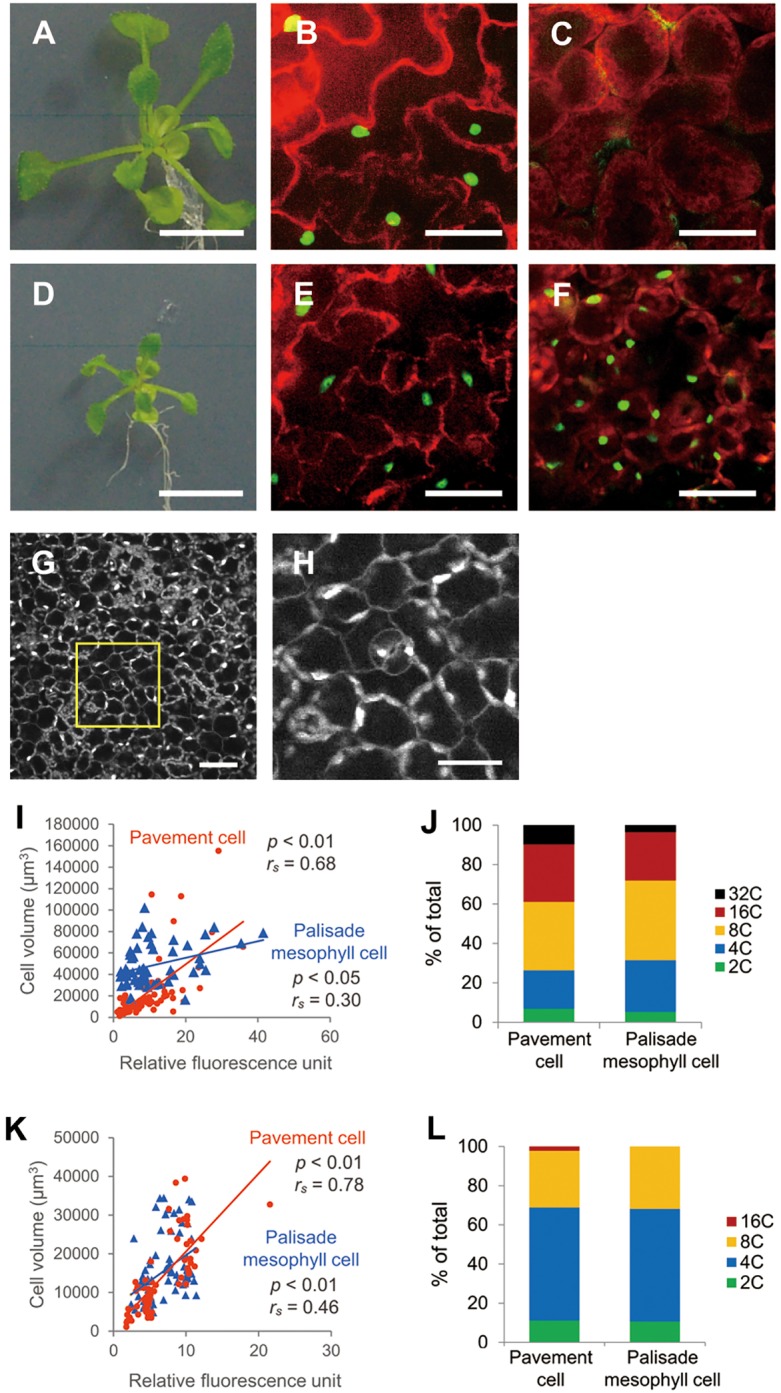
**The relationship between ploidy and cell volume of ATML1-induced palisade mesophyll cells.** (A-F) Effects of *ATML1* expression in proRPS5A-ATML1 upon β-estradiol treatment (D-F) and in the controls (A-C). (A,D) Plant seedlings treated with β-estradiol or DMSO (as a control) for 14 days, after a further 7 days of growth in MS medium. Fluorescence images of ATML1::NLS-3xGFP (green) and PI (red) in the epidermis (B,E) and in the palisade mesophyll (C,F) are shown. Scale bars: 10 mm (A,D) and 40 μm (B,C,E,F). (G,H) Ectopic guard cells. Panel H shows a magnified image of a part of the panel G, shown in a box. Scale bars: 50 μm (G) and 25 μm (H). (I,K) The relationship between relative fluorescence units and cell volume for individual pavement and palisade mesophyll cells in 21-day-old proRPS5A-ATML1 plants after 14 days of DMSO treatment (I) and β-estradiol treatment (K). *r_s_* indicates the Spearman rank correlation coefficient. A total of 68 pavement cells and 57 palisade mesophyll cells for I and 64 pavement cells and 73 palisade mesophyll cells for K were analysed. (J,L) Comparison of ploidy levels in pavement and palisade mesophyll cells in 21-day-old proRPS5A-ATML1 line after 14 days of DMSO (J) and β-estradiol (L) treatment.

We hypothesized that switching from the palisade mesophyll identity to the epidermis identity can alter the relationship between cell volume and ploidy level in inner cells of leaves. To test this hypothesis, we used an estradiol-inducible *ATML1*-expressing line harbouring proRPS5A::ATML1/pER8 and proATML1::nls-3xGFP (Takada et al., 2013; Fig. 3A-F). ARABIDOPSIS THALIANA MERISTEM LAYER 1 (ATML1) is a transcription factor that is specifically expressed in the outermost cell layer, and whose ectopic induction generates epidermal features such as stomatal guard cells and trichome-like cells in the mesophyll (Lu et al., 1996; Sessions et al., 1999). Because strong expression of *ATML1* induced a severe growth defect, we selected an appropriate induction level of *ATML1*, which allowed the transgenic line (line #35) to maintain a normal leaf layer structure (Takada et al., 2013). In the absence of β-estradiol, *ATML1* was expressed only in epidermal cells (Fig. 3B,C). After β-estradiol treatment, ectopic expression of *ATML1* was observed as GFP fluorescence in palisade mesophyll cells, indicating that the fate of the mesophyll cells had been changed towards that of epidermal cells (Fig. 3E-H). In addition, the mRNA transcript levels of inducible *ATML1* and the epidermal marker genes *FIDDLEHEAD* (*FDH*) and *ECERIFERUM 5* (*CER5*) were increased after treatment with β-estradiol (Fig. S2; Yephremov et al., 1999; Pruitt et al., 2000; Pighin et al., 2004). Under these conditions, 21-day-old seedlings showed reduced growth and generated only a few ectopic guard cells in the mesophyll tissue after 7 days of growth on the normal plate followed by 14 days of β-estradiol treatment (Fig. 3G,H).

We next compared the relationship between cell volume and ploidy level in palisade mesophyll cells before and after the induction of *ATML1*. Results were quite different for epidermal pavement cells (*P*<0.01, *r_s_=*0.68) and palisade mesophyll cells (*P*<0.05, *r_s_*=0.30) from dimethyl sulfoxide (DMSO)-treated control plants (Fig. 3I, Fig. S3, Table 1, Table S1), compared with WT plants (Fig. 2A,B). The control plants showed differential progression of endoreduplication between the epidermis and palisade tissues (Fig. 3J); higher levels of endoreduplication were observed in DMSO-treated control plants compared with WT plants (Fig. 2E,F). After induction of *ATML1*, progression of endoreduplication was similar in the different tissues (Fig. 3L). Interestingly, after induction of *ATML1* expression, the Spearman rank correlation coefficient of the palisade mesophyll cells (*P*<0.01, *r_s_=*0.46; Fig. 3K, Fig. S3, Table 1, Table S1) was significantly increased compared with cells without ATML1 (*r_s_*=0.30; Fig. 3I, Fig. S3, Table 1, Table S1). These results clearly demonstrate that cell identity plays an important role in the regulation of cell volume. It is noteworthy, considering our findings here, that the effect of constitutive *SIMEASE* (*SIM*) expression (which strongly enhances endoreduplication) on the increase in cell volume was also reported to be far stronger in the epidermis than in parenchymatous cells of leaves (see fig. 6E,F in Churchman et al., 2006). This variation might also be due to differences in the dependency of cell volume on ploidy level.

Previous analyses of endoreduplication were mainly performed using flow cytometry on whole leaves, which contained numerous mesophyll cells and a single layer of epidermal cells. By contrast, microscopy analyses on the correlation between ploidy levels and cell size have focused on the epidermis. These methods have been misleading and resulted in an overestimation of the relationship between ploidy levels and cell size in leaves. However, our results obtained using a tissue-clearing technique clearly show that cell volume control through endoreduplication differs in the epidermis and palisade tissues. The cell volume of palisade mesophyll cells appeared to be more conservative and stable against an increase in ploidy levels than epidermal cells. The robustness of the cell size in palisade mesophyll cells is probably derived from the physiological requirements of their important role in photosynthesis. The palisade layer is tightly packed, and thus each cell must be uniform in size. In addition, there must be a favourable ratio of cytosolic volume to total cell volume for efficient photosynthesis. Conversely, epidermal cells must be heterogeneous to ensure that the epidermal sheet is tolerant of tearing forces. That is, heterogeneous polyploidy and ploidy-dependent cell enlargement might offer mechanical resistance. Although cell proliferation in epidermis and mesophyll cells is coordinated by a signal from the mesophyll cells in a developing leaf primordia (Kawade et al., 2013), the final extension of the leaf blade by cell expansion is triggered by the epidermis (Zörb et al., 2015; Savaldi-Goldstein et al., 2007; Horiguchi and Tsukaya, 2011). These differential roles of the cell layers might also be associated with the different ploidy dependencies of cell size regulation. Indeed, the phenomenon of compensation (Tsukaya, 2008; Horiguchi and Tsukaya, 2011; Hisanaga et al., 2015), i.e. abnormally enhanced cell expansion in the palisade mesophyll cells, is typically independent of endoreduplication.

Previously, it was shown that pollen grains, petal epidermis and palisade mesophyll cells showed different ploidy dependencies for cell size in a whole-genome tetraploidization system (Breuer et al., 2007; Tsukaya, 2008). At this time, it is not possible to determine whether pollen, epidermal or palisade types represent the default for ploidy dependency of cell size regulation. However, the induction of epidermis identity in the inner cells of leaves results in a shift from the palisade mesophyll cell type relationship between cell volume and ploidy to the pavement cell type. Our results suggest that the weak palisade mesophyll cell type dependency on ploidy levels for cell enlargement is the default. In addition, although polyploidy might not be directly associated with cell volume, it is positively associated with genetic pathways (Tsukaya, 2013). This study highlights the need for precise tissue-specific analyses to understand the mechanism underlying the coordination between cell volume and organ size.

## MATERIALS AND METHODS

### Plant materials and growth conditions

pATML1::H2B-mGFP (Roeder et al., 2010), *rpt2a-1* (Sonoda et al., 2009), *rpt5a-4* (Sako and Yamaguchi, 2010), *cycA2;3* (Lu et al., 1996) and RPS5A-ATML1/pER8 (Takada et al., 2013) were described previously. *Arabidopsis thaliana* (Col-0) was grown on rockwool under a 16 h light:8 h dark cycle or in continuous light at 22°C, as described previously (Fujikura et al., 2007; Kozuka et al., 2005). For chemical induction of *ATML1* expression, plants were germinated on Murashige and Skoog (MS) agar plates [0.5×MS salts, 1% sucrose, and 0.8% agar (pH 5.8)] and grown in a plant chamber (CL-301, TOMY, Tokyo) under a 16 h light:8 h dark cycle at 22°C. After 7 days, seedlings were transferred to a MS agar plate containing 10 μM β-estradiol and grown for 14 days. β-estradiol was dissolved in dimethyl sulphoxide (DMSO) and the same volume of DMSO was added as a control.

### Flow cytometry

The first set of foliage leaves was collected for analysis of flow cytometry, as described previously (Kozuka et al., 2005) using EPICS XL and the BD Accuri C6 Flow Cytometer (Becton Dickinson). Protoplast isolation was performed as described previously (Wu et al., 2009).

### Technique for rendering leaves transparent

The first foliage leaves of 30-day-old seedlings were fixed in a mixture of ethanol and acetic acid (3:1) for 2 h. The samples were washed with 70% ethanol for 30 min and then incubated in sterile water for 5 min. After incubation in PBS for 10 min, the samples were stained with 5 μg/ml 4′,6-diamidino-2-phenylindole (DAPI) for 5 min and washed with PBS four times. To make the sections transparent, samples were incubated in 97% 2,2′-thiodiethanol (v/v in PBS buffer) for 30 min.

### Microscopy and data analysis

Seedlings were observed under an inverted fluorescence microscope (IX-81, Olympus) equipped with a confocal scanning unit (CSU-X1, Yokogawa) and a sCMOS camera (Neo 5.5 sCMOS, Andor) and a confocal microscope (FY1000, Olympus). One stack of 0.5 μm *z*-axis steps was collected. The cell volume was measured using ImageJ software (NIH; Fig. S2). To measure the intensity of integrated DAPI fluorescence, images were analysed using MetaMorph (Universal Imaging Corporation). The ploidy level was estimated optically according to the DAPI fluorescence intensity and normalized to the integrated intensity of guard cell nuclei. We fitted the cell volume of a palisade mesophyll cell to a cylinder. We calculated the cell volume by multiplication of the maximum cell area by the height along the *z*-axis (Fig. S4). Spearman rank coefficient tests were performed using R (v3.2.0) open software for statistical analysis.

### Quantitative real-time PCR

Seven-day-old seedlings of #35 lines were transferred to plates with or without 10 µM β-estradiol medium. After estradiol treatment, total RNA was extracted from shoot tissue of 10-day-old plants using the RNeasy Plant Mini kit (Qiagen). cDNA was synthesized using the Verso cDNA synthesis kit (Thermo Fisher Science). Quantitative real-time PCR was performed using Thunderbird SYBR qPCR mix (TOYOBO) and the Thermal Cycler Dice Real Time System III (Takara). The housekeeping gene encoding subunit A3 of protein phosphatase 2A was used for normalization in quantitative real-time PCR (Czechowski et al., 2005). PCR primer sequences are listed in Table S2.
